# Formation and Thermal Stability of Ordered Self-Assembled Monolayers by the Adsorption of Amide-Containing Alkanethiols on Au(111)

**DOI:** 10.3390/ijms24043241

**Published:** 2023-02-07

**Authors:** Young Ji Son, Jin Wook Han, Hungu Kang, Sicheon Seong, Seulki Han, Shoichi Maeda, Shunta Chikami, Tomohiro Hayashi, Masahiko Hara, Jaegeun Noh

**Affiliations:** 1 Department of Chemistry, Hanyang University, 222 Wangsimni-ro, Seongdong-gu, Seoul 04763, Republic of Korea; 2 Department of Chemistry, Korea University, 145 Anam-ro, Seongbuk-gu, Seoul 02841, Republic of Korea; 3 Department of Materials Science and Engineering, Tokyo Institute of Technology, 4259 Nagatsuta-cho, Midori-ku, Yokohama, Kanagawa 226-8503, Japan; 4 School of Materials and Chemical Technology, Tokyo Institute of Technology, 4259 Nagatsuta-cho, Midori-ku, Yokohama, Kanagawa 226-8503, Japan; 5 Research Institute for Convergence of Basic Science, Hanyang University, 222 Wangsimni-ro, Seongdong-gu, Seoul 04763, Republic of Korea

**Keywords:** self-assembled monolayers, *N*-(2-mercaptoethyl)heptanamide, decanethiol, adsorption, structure, thermal stability, amide group, hydrogen bonding, scanning tunneling microscopy

## Abstract

We examined the surface structure, binding conditions, electrochemical behavior, and thermal stability of self-assembled monolayers (SAMs) on Au(111) formed by *N*-(2-mercaptoethyl)heptanamide (MEHA) containing an amide group in an inner alkyl chain using scanning tunneling microscopy (STM), X-ray photoelectron spectroscopy (XPS), and cyclic voltammetry (CV) to understand the effects of an internal amide group as a function of deposition time. The STM study clearly showed that the structural transitions of MEHA SAMs on Au(111) occurred from the liquid phase to the formation of a closely packed and well-ordered β-phase via a loosely packed α-phase as an intermediate phase, depending on the deposition time. XPS measurements showed that the relative peak intensities of chemisorbed sulfur against Au 4f for MEHA SAMs formed after deposition for 1 min, 10 min, and 1 h were calculated to be 0.0022, 0.0068, and 0.0070, respectively. Based on the STM and XPS results, it is expected that the formation of a well-ordered β-phase is due to an increased adsorption of chemisorbed sulfur and the structural rearrangement of molecular backbones to maximize lateral interactions resulting from a longer deposition period of 1 h. CV measurements showed a significant difference in the electrochemical behavior of MEHA and decanethiol (DT) SAMs as a result of the presence of an internal amide group in the MEHA SAMs. Herein, we report the first high-resolution STM image of well-ordered MEHA SAMs on Au(111) with a (3 × 2√3) superlattice (β-phase). We also found that amide-containing MEHA SAMs were thermally much more stable than DT SAMs due to the formation of internal hydrogen networks in MEHA SAMs. Our molecular-scale STM results provide new insight into the growth process, surface structure, and thermal stability of amide-containing alkanethiols on Au(111).

## 1. Introduction

The surface and interface properties of metal surfaces can be readily tuned by the formation of closely packed and highly ordered self-assembled monolayers (SAMs) derived from organic molecules with chemically active anchoring groups and various backbone structures [1,2,3,4,5,6,7,8,9,10,11,12,13,14,15]. Hence, SAMs provide a versatile tool for the preparation of functional molecular thin films that can be applied to many practical applications, such as surface passivation [16], biointerfaces [17], biosensors [18], molecular photodiodes [19], batteries [20], and molecular electronic devices [21,22]. Among many organic thiols, *n*-alkanethiols with various alkyl chains are the most popular precursors because they easily form closely packed and well-ordered SAMs on gold with a high reproducibility. Therefore, alkanethiolate SAMs on gold have been extensively characterized to understand various fundamental aspects, such as adsorption behavior, growth kinetics, two-dimensional (2D) phase transitions, surface structures, and interface properties [1,2,8,14,16,17,23,24,25,26]. High-resolution scanning tunneling microscopy (STM) observations revealed that highly ordered alkanethiolate SAMs on Au(111) at saturation coverage have a (√3 × √3)R30° or (3 × 2√3) structure [1,2,8,26].

On the other hand, ϖ-functionalized alkanethiolate SAMs on gold have often been used to tune surface characteristics by changing the terminal functional groups of alkanethiols [1,2,27,28,29,30,31]. Amine-terminated SAMs are often utilized for the investigation of electron transfer reactions of specific proteins due to a strong binding affinity between the amine-terminal groups of SAMs and proteins [29]. An STM study showed that ethylene glycol-terminated alkanethiolate SAMs on Au(111) had very unique structural phases containing paired-row molecular domains and bright disordered regions, which were considerably different from alkanethiolate SAMs [14,28]. Electrochemical measurements demonstrated that anionic surfactants were strongly coupled to the ferrocene-terminal groups of alkanethiolate SAMs on gold electrodes via oxidation [31].

The structural stability of thiolate SAMs is of particular importance for applying SAMs to a variety of technological applications. Hence, the thermal and chemical stabilities of thiolate SAMs on gold surfaces have been extensively investigated by many research groups [1,7,10,22,23,24,32,33,34]. It was demonstrated that the hydrogen bonding between adsorbed molecules is a crucial factor with regard to controlling 2D self-assembled nanostructures [35,36,37]. It was also expected that SAMs of alkanethiols containing the internal amide group in the alkyl backbone on gold had more chemical stability than the corresponding alkanethiolate SAMs via buried interchain hydrogen bonding [37,38,39,40,41,42]. Interestingly, the charge transport behavior of alkanethiolate SAMs with an internal amide functional group were significantly influenced by amide functional groups and the conformation of molecules in the SAMs [43,44]. Reflection absorption infrared spectroscopy measurements showed that alkanethiolate SAMs containing amide groups had a strong amide-II frequency between the range of 1548 and 1557 cm^−1^, which strongly implied the formation of hydrogen bonding networks between the amide groups in alkanethiolate SAMs with an internal amide functional group [45,46,47]. Although the formation and surface structures of alkanethiolate SAMs with various alkyl chains on Au(111) have been thoroughly studied via STM from a molecular-scale viewpoint [1,2,13,24,25,26,33,34], very few STM reports exist regarding the surface structure and thermal stability of amide-containing alkanethiolate SAMs on Au(111) [40,41]. The first STM showed that 3-mercapto-*N*-nonylpropionamide (MNPA) SAMs had a slightly distorted (√3 × √3)R30° packing structure, with a linear molecular low [40]. In addition, MNPA SAMs showed very unique domain formation containing dark and bright domains with irregular shapes, which was due to a difference in the adsorption geometry of amide-containing alkyl backbones in SAMs [41].

**Figure 1:**
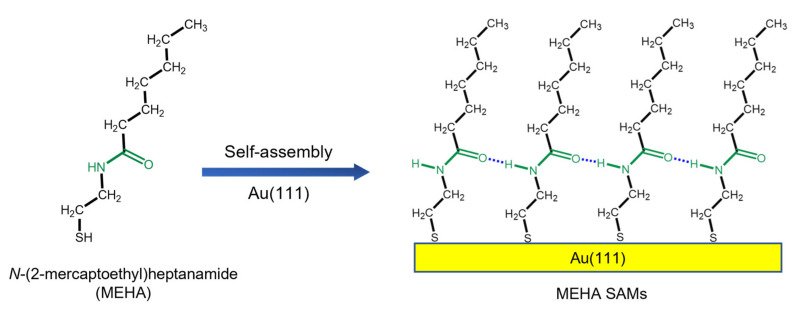
A chemical structure of MEHA with an internal amide functional group and a schematic view showing the formation of SAMs on Au(111) by the adsorption of MEHA molecules. The amide functional group in the chemical structure was indicated in the green color.

To deepen our understanding regarding the formation and thermal stability of amide-containing alkanethiolate SAMs on Au(111), we examined the surface structures, adsorption conditions, and reductive desorption behavior of SAMs on Au(111) using STM, XPS, and cyclic voltammetry (CV). Moreover, to understand the thermal stability more clearly, we compared the surface morphological changes of amide SAMs with those of alkanethiolate SAMs before and after annealing. For this purpose, we synthesized amide-containing alkanethiol (*N*-(2-mercaptoethyl)heptanamide, MEHA); the chemical structure of MEHA can be seen in Figure 1. MEHA has a shorter alkyl backbone (hexyl chains, C6) from the amide linker of the alkyl backbone, while MNPA has a longer alkyl backbone (nonyl chains, C9). In addition, the carbonyl groups of MEHA and MNPA exist opposite each other in the alkyl backbone. It was expected that the adsorption of MEHA on Au(111) generates the formation of chemisorbed monolayers by producing hydrogen ion in a solution (Figure 1) [13,15]. Herein, we report the first high-resolution STM image of MEHA SAMs on Au(111) with a (3 × 2√3) packing structure and the high thermal stability of MEHA SAMs compared to decanethiol (DT) SAMs. Our high-resolution STM will provide very meaningful information with regard to growth processes, surface structures, electrochemical behavior, and the thermal stability of amide-containing alkanethiols on Au(111).

## 2. Results and Discussion

### 2.1. Surface Structures of MEHA SAMs on Au(111) as a Function of Deposition Time

STM observations revealed that the amide functional group in the internal molecular backbone largely affected the domain formation and packing structure of SAMs [40,41]. Interestingly, the coexistence of randomly distributed dark and bright domains were observed for MNPA SAMs on Au(111) formed in a 1 mM EtOH solution at RT for 24 h, which resulted from different adsorption geometries of molecular backbones caused by the hydrogen bonding network in the SAMs. Despite many STM reports describing the growth processes of alkanethiolate SAMs on Au(111) as a function of deposition time [48,49], thus far, there have been no reports on those of internal amide-containing alkanethiol SAMs. In general, closely packed alkanethiolate SAMs were formed via several structural phases, such as a gas phase, striped phase, liquid phase, missing-low phase, and disordered phase [48,49,50]. To understand this issue regarding amide-containing alkanethiolate SAMs on Au(111), we investigated the surface features of MEHA SAMs in a 0.01 mM EtOH solution as a function of deposition time: 1 min, 10 min, and 1 h at RT.

**Figure 2:**
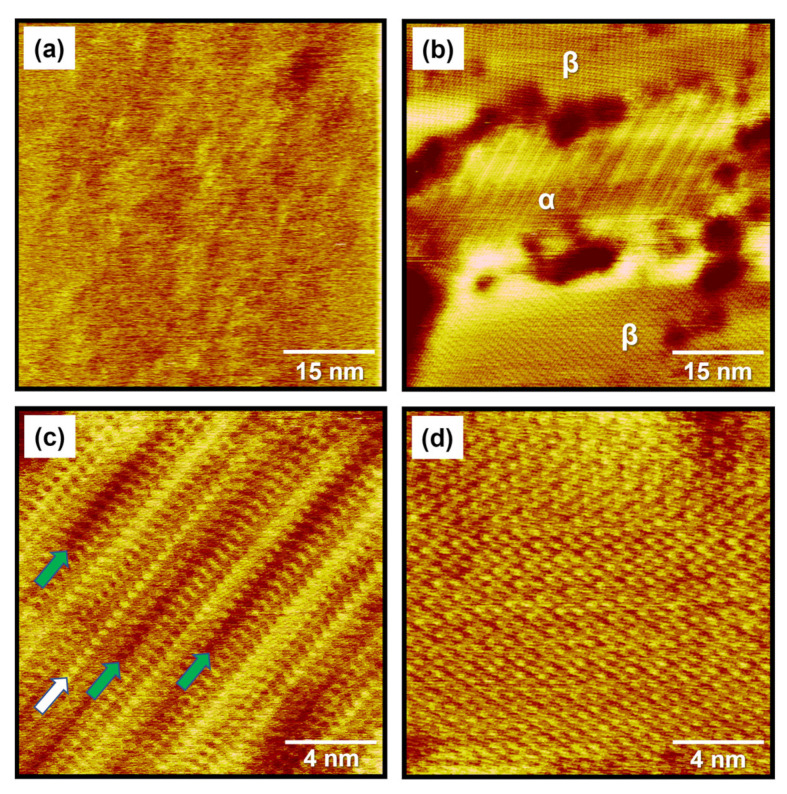
(**a**) STM image of MEHA SAMs on Au(111) formed in 0.01 mM EtOH solution at RT for 1 min. (**b**) STM image showing the coexistence of an ordered row phase (α phase) and closely packed and well-ordered phase (β phase) of MEHA SAMs on Au(111) formed in 0.01 mM EtOH solution at RT for 10 min. Magnified STM images showing (**c**) the α phase and (**d**) the β phase of MEHA SAMs on Au(111). Scan sizes of STM images were (**a,b**) 60 × 60 nm^2^ and (**c**,**d**) 15 × 15 nm^2^. Imaging conditions: (**a**) *V*_b_ = 450 mV and *I*_t_ = 550 pA, (**b**) *V*_b_ = 400 mV and *I*_t_ = 500 pA, (**c**) *V*_b_ = 300 mV and *I*_t_ = 600 pA, and (**d**) *V*_b_ = 300 mV and *I*_t_ = 20 pA.

**Figure 3:**
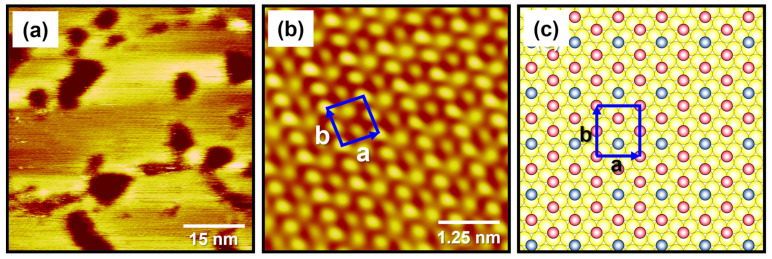
(**a**) STM image of MEHA SAMs on Au(111) formed in 0.01 mM EtOH at RT for 1 h. (**b**) High-resolution STM image of the well-ordered β phase of MEHA SAMs on Au(111). (**c**) A proposed structural model of MEHA SAMs on Au(111). Scan sizes of STM images were (**a**) 60 × 60 nm^2^ and (**b**) 5 × 5 nm^2^. Imaging conditions: (**a**) *V*_b_ = 620 mV and *I*_t_ = 550 pA and (**b**) *V*_b_ = 280 mV and *I*_t_ = 725 pA.

After deposition for 1 min, the STM image in Figure 2a shows that there is no observable structural order of MEHA SAMs over the entire Au(111) surface. It was attributed to the formation of a liquid phase on Au(111) that has often been observed at a low surface coverage during the initial growth stage of alkanethiolate SAMs at a low surface coverage prior to the formation of a condensed phase [48,49,50]. This result is strongly supported by our XPS and CV results (discussed later). However, when the deposition time increased from 1 to 10 min, the surface structures of MEHA SAMs on Au(111) drastically changed from the liquid phase to the 2D ordered phase, as shown in Figure 2b–d. Interestingly, we found the coexistence of two structural phases: an ordered row structure (α-phase) and well-ordered and closely packed structure (β-phase), as shown in Figure 2b. In addition, several vacancy islands (VIs) with a monatomic depth of 2.5 Å (dark trench areas) were observed on the surface, which were well-known intrinsic characteristics that appeared as a result of the formation of chemisorbed monolayers on Au(111) surfaces by the adsorption of S- or Se-containing molecules [1,2,3,4,5,11,14,48,49,50]. Note that the VIs were also fully covered with a monolayer, not a bare Au(111) surface, as already demonstrated via high-resolution STM observations for thiolate or selenolate SAMs on Au(111) [3,5,6]. It was found that most VIs on the Au(111) surfaces existed near domain boundaries between the α- and β-phases. This result implied that the movement of VIs to domain boundary regions was driven by the optimization of van der Waals interactions between molecular backbones in the ordered domains. The high-resolution STM image in Figure 2c shows that the α-phase was composed of ordered molecular rows with various imaging contrasts; there were no periodic patterns between the rows. Therefore, we were not able to assign adlayer structures corresponding to the α-phase. The distance between molecular spots along the row indicated by the white arrow was measured to be 5.1 ± 0.2 Å, which was ~√3 times against the diameter of Au atom with a 2.89 Å. Several dark rows indicated by green arrows were also observed, which were considered to be missing rows. Similar structures usually appeared prior to the formation of closely packed SAMs [49,50] or after the desorption of adsorbed molecules in the closely packed SAMs resulting from thermal annealing [24,51] or long-term storage in pure solvent [33] and UHV conditions [34]. Therefore, the α-phase was considered to be an intermediate phase that could appear before the formation of closely packed monolayers. The STM image in Figure 2d shows a closely packed and well-ordered β-phase of MEHA SAMs on Au(111). After a longer deposition for 1 h, we observed the same β-phase with much better STM resolution (Figure 3b). Therefore, structural details for this phase will be discussed later. From our STM results showing the existence of two mixed phases (the α- and β-phases) on Au(111), it was clear that a deposition time of 10 min was not sufficient for the formation of a uniform well-ordered phase for MEHA SAMs.

When the deposition time increased from 10 min to 1 h, the α-phase of MEHA SAMs on Au(111) completely disappeared and a β-phase solely formed, as shown in Figure 3a. This meant that the β-phase was more energetically favorable than the α-phase. Structural transitions from the α-phase to the β-phase were mainly driven by an increase in surface coverage. The molecularly resolved STM image in Figure 3b clearly shows that the β-phase of MEHA SAMs on Au(111) have closely packed and well-ordered structures. This is the first high-resolution STM image of MEHA SAMs on Au(111). Based on this high-resolution STM image, the lattice parameters of a rectangular unit cell (small arrows squire in Figure 3b,c) were extracted: a = 8.7 ± 0.2 Å = 3a_h_, b = 9.9 ± 0.2 Å = 2√3a_h_. Note that a_h_ is the spacing between gold atoms, 2.89 Å. Figure 3c shows the proposed structural model of the β-phase of MEHA SAMs on Au(111). Therefore, the highly ordered β-phase for MEHA SAMs on Au(111) was assigned to be a (3 × 2√3) packing structure, which was the same structure as usually observed for well-ordered and closely packed alkanethiolate SAMs [2,26,29,33]. On the other hand, a previous STM study revealed that MNPA SAMs with an internal amide group formed in a 1 mM EtOH solution at RT for 5 days were composed of two dominant phases with a slightly distorted (√3 × √3) structure and a zig-zag packing structure (adsorbed molecules were not clearly visualized via STM) [40]. From our high-resolution STM study, we clearly demonstrated that, although MEHA SAMs on Au(111) had a different packing structure compared to MNPA SAMs, MEHA and MNPA SAMs possessed the same average areal density of 21.5 Å^2^/molecule as in the cases of closely packed alkanethiolate SAMs. From these results, it was suggested that the formation of an internal hydrogen bonding network in amide-containing alkanethiolate SAMs did not significantly affect the 2D packing structure of SAMs. 

### 2.2. Binding Conditions of MEHA SAMs on Au(111) as a Function of Deposition Time

**Figure 4:**
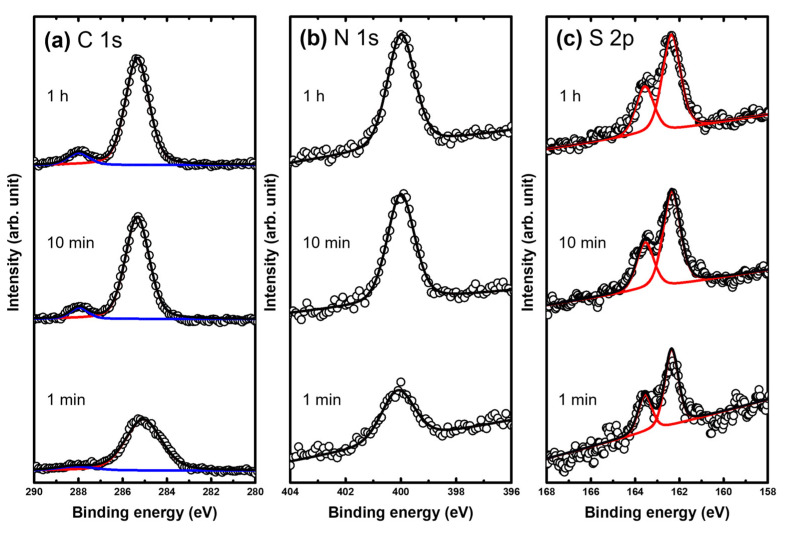
The (**a**) C 1s, (**b**) N 1s, and (**c**) S 2p regions of the XPS spectra of MEHA SAMs on Au(111) formed in 0.01 mM EtOH solution at RT for 1 min, 10 min, and 1 h. The dark lines correspond to the normalized XPS peaks and the colored lines correspond to the fitted XPS peaks.

The binding conditions of MEHA SAMs on Au(111) as a function of deposition time (1 min, 10 min, and 1 h) were investigated via XPS to understand the growth processes of the SAMs. Figure 4 shows high-resolution XPS spectra in the region of C 1s, N 1s, and S 2p for MEHA SAMs on Au(111) formed in a 0.01 mM EtOH solution at RT for 1 min, 10 min, and 1 h, respectively. Two C 1s peaks were observed, as shown in Figure 4a. The C 1s peak corresponding to an alkyl chain with a strong intensity was observed at around 285.2 eV, while the carbonyl carbon peak in the amide group was observed at a higher binding energy of around 288.1 eV. On the other hand, the full width at half maximum (FWHM) values for the N 1s (S 2p) peaks of MEHA SAMs that formed after deposition for 10 min or 1 h were found to be 1.24 eV (1.36 eV) and 1.23 eV (1.33 eV), which were much narrower than those for MEHA SAMs with 1.85 eV (2.07 eV) formed after deposition for 1 min, respectively. We considered that the wider FWHM for MEHA SAMs for 1 min was due to the presence of randomly oriented molecules in the liquid phase, while the narrower FWHM for MEHA SAMs for 10 min or 1 h was due to the formation of a well-ordered crystalline phase, as demonstrated via STM (Figure 2 and Figure 3). In addition, the N 1s peak for all SAM samples was observed at 400.0 eV, which originated from the nitrogen of the amide functional group. Similar XPS peaks in the region of C 1s and N 1s were also observed for MNPA SAMs [41], implying the presence of an internal amide group in MEHA SAMs on Au(111). Note that the S 2p peak appeared as a doublet consisting of 2p_3/2_ and 2p_1/2_ peaks in a 2:1 intensity ratio caused by spin-orbital splitting [2,7,26,34,51]. Doublet S 2p XPS spectra of MEHA SAMs on Au(111) were observed at 162.3 eV (2p_3/2_) and 163.5 eV (2p_1/2_), as shown in Figure 4c. This result meant that there existed only one adsorption state of sulfur in MEHA SAMs on Au(111) regardless of deposition time. The peaks could be assigned to chemisorbed sulfurs, implying that MEHA SAMs formed via chemical interactions between the sulfur anchoring group and Au(111) surface. Similar chemisorbed sulfur peaks at around 162 eV were usually observed for various thiolate SAMs on gold [2,7,26,34,52]. The relative peak intensities of chemisorbed sulfur against Au 4f for MEHA SAMs on Au(111) formed after deposition for 1 min, 10 min, and 1 h were calculated to be 0.0022, 0.0068, and 0.0070, respectively. The adsorption amount of chemisorbed sulfur for MEHA SAMs formed after deposition for 1 min was ~3 times lower than that formed after deposition for 10 min or 1 h. Therefore, it was hard to form the solid phase of MEHA SAMs after a short deposition time of 1 min. This XPS result was in good agreement with the STM result showing the liquid phase, as can be seen in Figure 2a. After deposition for 10 min, the adsorption amount of chemisorbed sulfur increased drastically by more than ~3 times, resulting in the formation of a solid phase for SAMs showing ordered α- and β-phases. On the other hand, after deposition for 1 h, the adsorption amount of chemisorbed sulfur slightly increased, resulting in the formation of a uniform β-phase on the entire Au(111) surface. Based on the STM and XPS results, it was considered that the formation of a β-phase as a dominant structure was due to an increase in the adsorption of chemisorbed sulfur and a structural rearrangement of the molecular backbone to maximize lateral interactions during a longer deposition time. Our STM results showing the structural transition from the liquid phase to the solid phase (the ordered α- and β-phases) was strongly supported by the XPS results.

### 2.3. Comparative Study of Reductive Desorption (RD) Behavior for MEHA and DT SAMs on Au(111)

**Figure 5:**
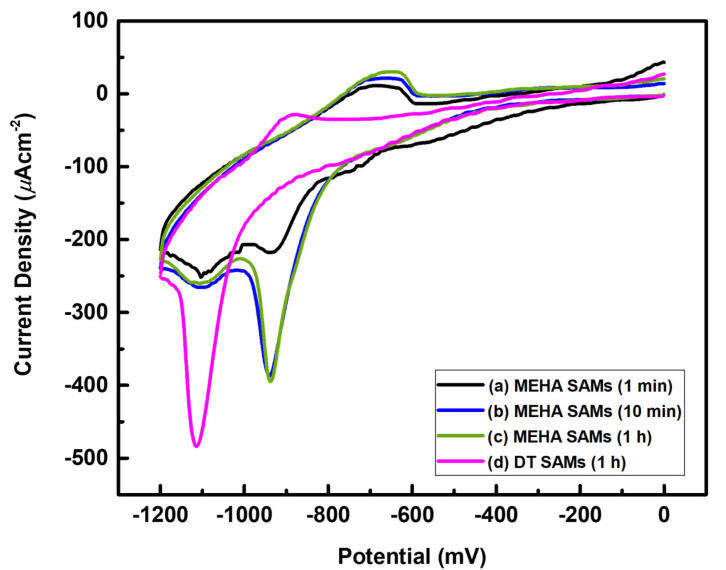
CVs of RD for MEHA SAM-modified Au(111) electrodes formed in 0.01 mM EtOH at RT as a function of immersion time: (**a**) 1 min, (**b**) 10 min, and (**c**) 1 h. (**d**) CVs of RD for DT SAM-modified Au(111) electrodes formed in 0.01 mM EtOH at RT for 1 h.

It has been reported that the position and shape of RD peaks for SAM-covered Au electrodes were significantly influenced by the binding affinity between the anchoring group and the Au surface, the magnitude of van der Waals interactions between adsorbed molecules, and the degree of structural order of the SAMs [14,31,41,52,53,54]. To examine the electrochemical behavior of MEHA SAMs formed at different deposition times on Au(111), we measured the CVs of RD for MEHA SAM-modified Au electrodes, as shown in Figure 5. Moreover, to understand the effect of an internal amide group in MEHA SAMs with regard to electrochemical behavior, we also measured the CVs of RD for DT SAM-modified Au electrodes prepared in a 0.01 mM EtOH solution at RT for 1 h (Figure 5). We found that the RD behavior of MEHA SAMs formed after deposition for 1 min was markedly different from those that formed after deposition for 10 min and 1 h, as shown in Figure 5a–c. Two RD peaks for MEHA SAMs formed after deposition for 1 min were observed at around −935 and −1102 mV, whereas those for MEHA SAMs formed after deposition for 10 min and 1 h were observed at around −940 and −1104 mV. The first RD peak MEHA SAMs, as a main peak that formed after deposition for 10 min and 1 h, were shifted to a slightly more negative potential with sharp and strong intensities compared to that formed after deposition for 1 min (broad and weak intensity). This remarkable difference in electrochemical behavior should be due to the formation of highly ordered MEHA SAMs resulting from an increase in surface coverage, as demonstrated by our STM and XPS study. It was demonstrated that, when van der Waals interactions in the SAMs increased, the structural quality of the SAMs was significantly enhanced. Hence, RD peaks were also shifted to a more negative potential [14,27,31,41,52,53]. Moreover, similar second RD peaks with a broad hump between −1070 and −1130 mV were observed for all SAM samples regardless of deposition time. Although it is difficult to exactly assign the origin of this peak at present, it was proposed that similar peaks with broad humps were probably due to the desorption of sulfur atoms on Au surfaces resulting from S–C bond cleavage during electrochemical reactions or caused by the adsorption of sulfur impurities present in the thiol solution [54].

**Figure 6:**
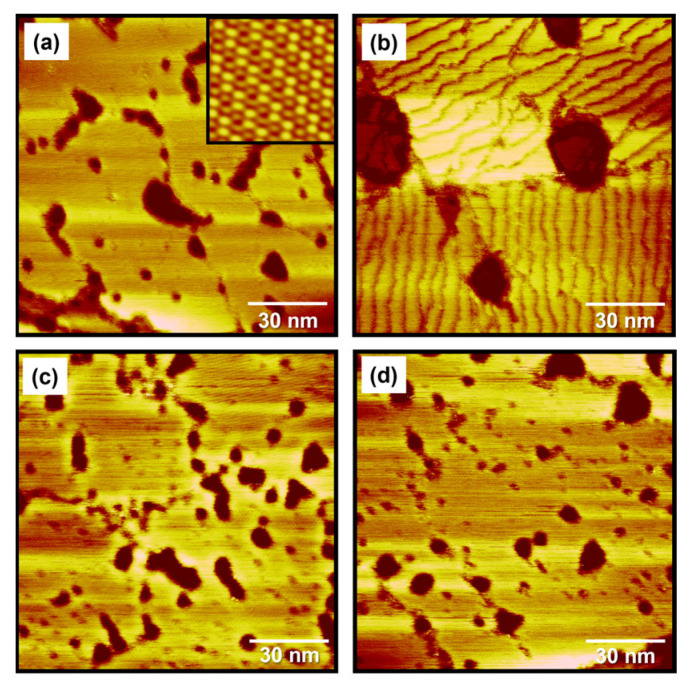
(**a,b**) STM images of pre-covered DT SAMs on Au(111) formed in 0.01 mM EtOH solution at RT for 1 h (**a**) before and (**b**) after thermal annealing at 373 K for 1 h. The high-resolution inset STM image (5 × 5 nm^2^) in Figure 6a shows that DT SAMs have a (3 × 2√3) packing structure. (**c**,**d**) STM images of MEHA SAMs on Au(111) formed in 0.01 mM EtOH solution at RT for 1 h (**c**) before and (**d**) after thermal annealing at 373 K for 1 h. Scan sizes of all STM images were 120 × 120 nm^2^, respectively. Imaging conditions: (**a**) *V*_b_ = 560 mV and *I*_t_ = 300 pA, (**b**) *V*_b_ = 500 mV and *I*_t_ = 350 pA, (**c**) *V*_b_ = 610 mV and *I*_t_ = 450 pA, and (**d**) *V*_b_ = 500 mV and *I*_t_ = 550 pA.

It was found that the main RD peak for DT SAMs was observed at −1115 mV, which was shifted to a much more negative potential by 75 mV compared to MEHA SAMs with −940 mV. STM observations revealed that DT SAMs have a (3 × 2√3) packing structure (Figure 6a) similar to MEHA SAMs, suggesting that both SAMs have the same molecular packing density. Therefore, the large difference in electrochemical behavior could be attributed to the presence of an internal amide group of MEHA SAMs. The amide group has electron withdrawing character, which acts as a good electron acceptor, such that the MEHA molecules in the monolayer can easily be desorbed from the Au surface via facile reductive reactions at the Au electrodes. Similar RD processes for MNPA SAMs on Au(111) were also observed between the range of −920 and −950 mV, which depended on the structural quality of amide-containing SAMs [41].

### 2.4. Comparative Study of Thermal Stability for MEHA and DT SAMs on Au(111)

**Figure 7:**
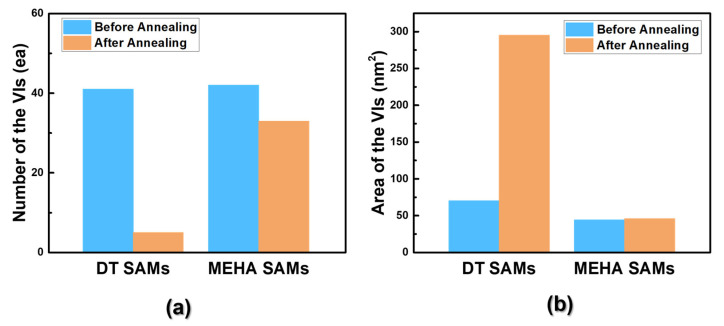
A diagram showing a remarkable difference in the (**a**) number and (**b**) area of VIs to the total surface area for DT and MEHA SAMs before and after thermal annealing.

The thermal stability of organic thiol SAMs on gold surfaces is a very important issue for SAM-based practical applications, such that many studies have been conducted to understand their thermal stability [1,6,10,24,51,55,56,57]. A TDS study revealed that alkanethiolate SAMs on Au(111) surfaces were initially desorbed as in the form of a dialkyl disulfide species (RSSR^+^) via a dimerization reaction of sulfur anchoring groups at a low temperature of 350 K, while alkanethiolate (RS^+^) species formed via the bond cleavage of RS-Au were initially desorbed at a high temperature of 410 K [10,55]. Molecular-scale STM observations showed that structural changes in alkanethiolate SAMs occurred, resulting from the desorption of adsorbed molecules during thermal annealing at an elevated temperature under air or UHV conditions [24,51,56]. In contrast to many works for alkanethiolate SAMs, there have been few reports describing the thermal stability of internal amide-containing alkanethiolate SAMs on gold [42]. Previous TDS measurements showed that hydrogen bonding networks between amide groups in alkanethiolate SAMs could enhance the thermal stability of SAMs [42]. To understand the effects of an internal amide group on the thermal stability of SAMs from a molecular-scale viewpoint, we examined and compared the surface morphology of pre-covered MEHA and DT SAMs on Au(111) formed in 0.01 mM EtOH at RT for 1 h before and after thermal annealing under air at 373 K for 1 h (Figure 6). The STM image in Figure 6a shows the surface structures of pre-covered DT SAMs with a well-ordered (3 × 2√3) structure (the inset 5 nm × 5 nm STM image in Figure 6a). A number of VIs were distributed on the entire Au(111) surface. Nearly identical surface structures were also observed for DT SAMs prepared using different SAM preparation conditions (2 mM EtOH solution and deposition for 48 h) [24], suggesting that our SAM preparation conditions (0.01 mM EtOH solution and deposition for 1 h) were enough for the formation of closely packed and well-ordered monolayers). However, the surface structures of pre-covered DT SAMs changed drastically after thermal annealing at 373 K for 1 h, as shown in Figure 6b. Many missing-row defects (dark rows) in the ordered domains appeared due to the desorption of adsorbed thiolate molecules. Moreover, the number of VIs largely decreased and the size of VI became larger after thermal annealing to maximize van der Waals interactions between alkyl chains resulting from the well-known Ostwald ripening process, as suggested by previous literatures [57]. Similar structural changes in the ordered domains and VIs after thermal annealing were also observed using STM for various alkanethiolate SAMs on Au(111) [24,51,56,57]. In contrast to alkanethiolate SAMs, the surface structures (ordered domains and VIs) of MEHA SAMs were nearly the same before (Figure 6c) and after (Figure 6d) thermal annealing. Based on the STM images of DT and MEHA SAMs on Au(111) (Figure 6), the number and area of VIs to the total surface area were analyzed. We found that the ordered domains and the number and size of VIs for DT SAMs changed significantly, but those for MEHA SAMs were almost the same, implying that MEHA SAMs had higher thermal stability compared to DT SAMs (Figure 7). Our results were strongly supported by the results of external reflective IR spectrometry showing that amide-containing SAMs can be stabilized via hydrogen bonding between the inner-functionalized amide groups of alkanethiols [37,38,39]. From our molecular-scale STM study, we clearly demonstrated that MEHA SAMs were thermally much more stable than alkanethiolate SAMs due to the formation of internal hydrogen networks in MEHA SAMs (see Figure 1).

## 3. Materials and Methods

### 3.1. Chemicals and Au(111) Substrates

MEHA was simply synthesized by the reaction of heptanoic acid (Sigma Aldrich, St.Louis, MO, USA) and 2-aminoethanethiol (Tokyo Chemical Industry, Tokyo, Japan) according to a reported literature method [58] and was confirmed via ^1^H NMR. DT [CH_3_(CH2)_9_SH] was purchased from Tokyo Chemical Industry, Japan. The synthetic details can be found in the Supporting Information (Figures S1 and S2). Single-crystal Au(111) substrates for SAMs were prepared by the thermal deposition of gold on mica under UHV conditions of ~10^−6^ Pa at 623 K [6].

### 3.2. Preparation of MEHA and DT SAMs

For cleaning the bare Au(111) substrate prior to SAM deposition, the gold substrates were annealed in a furnace at 733 K for 4 h and immediately quenched in N_2_-bubbled ethanol (EtOH) solvent. MEHA SAMs were prepared by immersion of the Au(111) substrate in a 0.01 mM EtOH solution of MEHA for a desired period (1 min, 10 min, and 1 h) at RT. A schematic view of the formation of MEHA SAMs through hydrogen bonding networks can be seen in Figure 1. The resulting MEHA SAMs on Au(111) were removed from solution, immediately rinsed with pure EtOH solvent, and dried with an N_2_ stream. To compare the thermal stability of MEHA and DT on Au(111), pre-covered DT SAMs were also prepared after 1 h deposition in a 0.01 mM EtOH solution of DT. To compare the thermal stability of pre-covered MEHA and DT SAMs, both SAMs were annealed at 373 K for 1 h.

### 3.3. Characterizations

STM measurements were carried out using a NanoScope E (Veeco, Santa Barbara, CA, USA) with a Pt/Ir (80:20) tip in air. Typical imaging parameters were used for the bias voltages (*V*_b_) of 200–700 mV (sample positive) and the tunneling currents (*I*_t_) of 300–800 pA. XPS measurements were performed with a Theta Probe (Thermo Fisher Scientific, Waltham, UK) with a monochromatic Al Kɑ radiation source (E = 1486.6 eV). The reductive desorption peaks for MEHA SAM-modified Au(111) electrodes were obtained using a BAS-100 electrochemical instrument in N_2_-bubbled 0.1 M KOH solution at a scan rate of 400 mV/sec.

## 4. Conclusions

The surface structures, binding conditions, electrochemical behavior, and thermal stability of MEHA SAMs on Au(111) were examined as a function of deposition time using STM, XPS, and CV. STM observations clearly revealed that the phase transitions of MEHA SAMs on Au(111) occurred from the liquid phase to a closely packed and well-ordered β-phase via an ordered-row α-phase as an intermediate phase as the deposition time increased from 1 min to 1 h. We reported the first high-resolution STM image of the β-phase for MEHA SAMs on Au(111) that formed after deposition for 1 h, which is described as a (3 × 2√3) packing structure. XPS measurements showed that the relative peak intensities of chemisorbed sulfur against Au 4f for MEHA SAMs on Au(111) that formed after deposition for 1 min, 10 min, and 1 h were calculated to be 0.0022, 0.0068, and 0.0070, respectively. Based on the STM and XPS results, we considered that the formation of the β-phase was due to an increase in the adsorption of chemisorbed sulfur and a structural rearrangement of the molecular backbone to maximize lateral interactions during longer deposition periods. CV measurements showed that the main RD peak for DT SAMs was observed at −1115 mV, which was shifted to a much more negative potential compared to MEHA SAMs with −940 mV. The less negative potential of MEHA SAMs was due to the amide group in MEHA SAMs that acted as a good electron acceptor, such that the MEHA molecules in the monolayer could easily be desorbed from the Au surface via facile reductive reactions. Moreover, our molecular-scale STM observations clearly demonstrated that MEHA SAMs were thermally much more stable than alkanethiolate SAMs due to the formation of internal hydrogen networks in MEHA SAMs.

## Data Availability

The data are included within this article and Supplementary Materials.

## Supplementary Materials

The following supporting information can be downloaded at https://www.mdpi.com/article/10.3390/ijms24043241/s1.
