# Supplementary material for: Formation and Thermal Stability of Ordered Self-Assembled Monolayers by the Adsorption of Amide-Containing Alkanethiols on Au(111)
Source: Int J Mol Sci. 2023 Feb 7;24(4):3241. doi: 10.3390/ijms24043241 (PMC9967528; doi:10.3390/ijms24043241)
Supplement: Supplementary file 1 [file ijms-24-03241-s001.zip › ijms-2187238-supplementary.pdf]

SUPPLEMENTARY MATERIALS

Formation and Thermal Stability of Ordered Self-Assembled Monolayers by Adsorption of Amide-Containing Alkanethiols on Au(111)

Young Ji Son[1,†], Jin Wook Han[1,†], Hungu Kang[1,2], Sicheon Seong[1], Seilki Han[1], Shoichi Maeda[3], Shunta Chikami[3], Tomohiro Hayashi[3], Masahiko Hara[4], Jaegeun Noh[1,5,*]

[1]Department of Chemistry, Hanyang University, 222 Wangsimni-ro, Seongdong-gu, Seoul 04763, Korea

[2]Department of Chemistry, Korea University, 145 Anam-ro, Seongbuk-gu, Seoul 02841, Korea

[3]Department of Materials Science and Engineering, School of Materials and Chemical Technology, Tokyo Institute of Technology, 4259 Nagatsuta-cho, Midori-ku, Yokohama, Kanagawa 226-8503, Japan

[4] Department of Electronic Chemistry, Tokyo Institute of Technology, 4259 Nagatsuta, Midoriku, Yokohama 226-8502, Japan

[5]Research Institute for Convergence of Basic Science, Hanyang University, 222 Wangsimni-ro, Seongdong-gu, Seoul 04763, Korea

[*] Correspondence: jgnoh@hanyang.ac.kr

[†] These authors contributed equally to this work.

1. Synthesis of N-(2-mercaptoethyl)heptanamide (MEHA)

1.1. Materials

Heptanoic acid (≥99.0%, Sigma-Aldrich), N-hydroxysuccinimide (NHS; ≥98.0%, TCI), N, N'-dicyclohexylcarbodiimide (DCC; ≥98.0%, TCI), Dichloromethane (DCM; ≥99.8%, Sigma-Aldrich), 2-Aminoethanethiol (≥95.0%, TCI), and triethylamine (≥99.0%, Sigma-Aldrich) were purchased and used without further purification.

1.2. Synthesis of MEHA

2,5-dioxopyrrolidin-1-yl heptanoate: At 0 °C, DCC (6.190 g, 30 mmol) was added to a dioxane solution (500 mL) containing heptanoic acid (3.9056 g, 30 mmol) and NHS (3.452 g, 30 mmol), and the mixture was stirred at 20 °C overnight. 2,5-dioxopyrrolidin-1-yl heptanoate, a white solid obtained by evaporating the reaction mixture, was then used for the subsequent procedure without further purification.

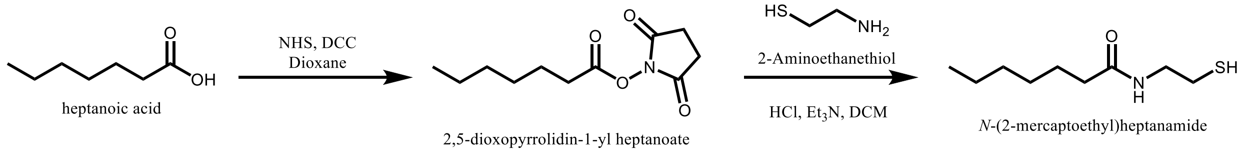

Figure S1. Synthetic schematic illustration of MEHA

N-(2-mercaptoethyl)heptanamide (MEHA): 2,5-dioxopyrrolidin-1-yl heptanoate and triethylamine (8.31 mL, 60 mmol) were added to a DCM solution (350 mL) and stirred for 4 h at 20 °C.

The reaction mixture was washed twice in 100 mL of 1 N aqueous HCl, dried over Na_2SO_4, and then evaporated to dryness. The residue was purified with an eluent (hexane: EA=1:1) over silica gel, and the second layer was isolated from the column and evaporated to produce MEHA as a white solid (1.4114 g, 24.9 %). ^{1}H NMR (400 MHz, $CDCl_3$): δ (ppm) 5.832 (br, 1H), 3.463-3.416 (q, 2H), 2.212-2.174 (t, 2H), 1.672-1.598 (m, 2H), 1.356-1.242 (m, 7H), 0.902-0.868 (t, 3H).

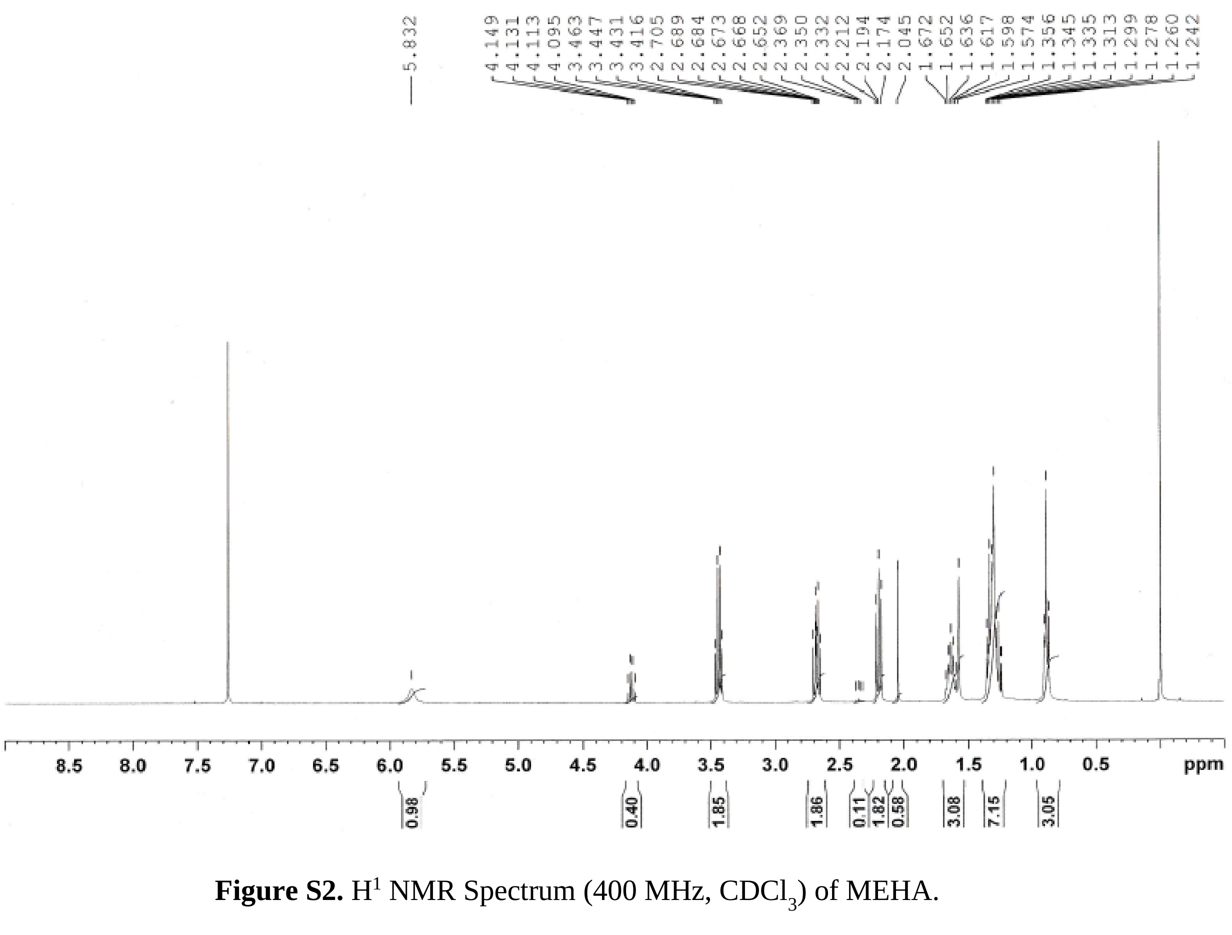

Figure S2. H^1 NMR Spectrum (400 MHz, $CDCl_3$) of MEHA.
